# Malaria Burden and Artemisinin Resistance in the Mobile and Migrant Population on the Thai–Myanmar Border, 1999–2011: An Observational Study

**DOI:** 10.1371/journal.pmed.1001398

**Published:** 2013-03-05

**Authors:** Verena I. Carrara, Khin Maung Lwin, Aung Pyae Phyo, Elizabeth Ashley, Jacher Wiladphaingern, Kanlaya Sriprawat, Marcus Rijken, Machteld Boel, Rose McGready, Stephane Proux, Cindy Chu, Pratap Singhasivanon, Nicholas White, François Nosten

**Affiliations:** 1 Shoklo Malaria Research Unit, Mae Sot, Thailand; 2 Mahidol Oxford University Research Unit, Bangkok, Thailand; 3 Centre for Tropical Medicine, University of Oxford, Oxford, United Kingdom; 4 Department of Obstetrics, University Medical Center Utrecht, Utrecht, The Netherlands; 5 Academic Medical Center, Amsterdam, The Netherlands; 6 Faculty of Tropical Medicine, Mahidol University, Bangkok, Thailand; Médecins Sans Frontières, South Africa

## Abstract

**Background:**

The Shoklo Malaria Research Unit has been working on the Thai–Myanmar border for 25 y providing early diagnosis and treatment (EDT) of malaria. Transmission of *Plasmodium falciparum* has declined, but resistance to artesunate has emerged. We expanded malaria activities through EDT and evaluated the impact over a 12-y period.

**Methods and Findings:**

Between 1 October 1999 and 30 September 2011, the Shoklo Malaria Research Unit increased the number of cross-border (Myanmar side) health facilities from two to 11 and recorded the number of malaria consultations. Changes in malaria incidence were estimated from a cohort of pregnant women, and prevalence from cross-sectional surveys. In vivo and in vitro antimalarial drug efficacy were monitored. Over this period, the number of malaria cases detected increased initially, but then declined rapidly. In children under 5 y, the percentage of consultations due to malaria declined from 78% (95% CI 76–80) (1,048/1,344 consultations) to 7% (95% CI 6.2–7.1) (767/11,542 consultations), *p<*0.001. The ratio of *P. falciparum/P. vivax* declined from 1.4 (95% CI 1.3–1.4) to 0.7 (95% CI 0.7–0.8). The case fatality rate was low (39/75,126; 0.05% [95% CI 0.04–0.07]). The incidence of malaria declined from 1.1 to 0.1 episodes per pregnant women-year. The cumulative proportion of *P. falciparum* decreased significantly from 24.3% (95% CI 21.0–28.0) (143/588 pregnant women) to 3.4% (95% CI 2.8–4.3) (76/2,207 pregnant women), *p<*0.001. The in vivo efficacy of mefloquine-artesunate declined steadily, with a sharp drop in 2011 (day-42 PCR-adjusted cure rate 42% [95% CI 20–62]). The proportion of patients still slide positive for malaria at day 3 rose from 0% in 2000 to reach 28% (95% CI 13–45) (8/29 patients) in 2011.

**Conclusions:**

Despite the emergence of resistance to artesunate in *P. falciparum*, the strategy of EDT with artemisinin-based combination treatments has been associated with a reduction in malaria in the migrant population living on the Thai–Myanmar border. Although limited by its observational nature, this study provides useful data on malaria burden in a strategically crucial geographical area. Alternative fixed combination treatments are needed urgently to replace the failing first-line regimen of mefloquine and artesunate.

*Please see later in the article for the Editors' Summary*

## Abstract 2

Francois Nosten and colleagues evaluate malaria prevalence and incidence in the mobile population on the Myanmar side of the border with Thailand between 1999 and 2011, and also assess resistance to artemisinin.

## Editors' Summary

**Background:**

According to latest figures, the World Health Organization estimates that there are over 200 million cases of malaria each year, with over three-quarters of a million deaths. Several *Plasmodium* parasites cause malaria (the most serious being *Plasmodium falciparum*) and are transmitted to people through the bites of infected night-flying mosquitoes. Malaria transmission can be prevented by using insecticides to control the mosquitoes and by sleeping under insecticide-treated bed nets. However, in Southeast Asia the effectiveness of these measures is limited. Treating infected people with antimalarial drugs, particularly with artemisinin-based combination treatments (ACTs), is a key strategy in reducing the deaths and disability caused by malaria. However, progress is now threatened by the emergence in Southeast Asia of *P. falciparum* isolates that are resistant to artesunate (a common component of ACT). This development is concerning, as resistance to the artemisinin family of drugs, of which artesunate is a member, could trigger a resurgence in malaria in many parts of the world and compromise the progress made in the treatment of severe malaria.

**Why Was This Study Done?:**

*P. falciparum* resistance to artemisinin has been confirmed in the area around the border between Thailand and Myanmar. Malaria control in this border area is particularly challenging, as there is a reservoir of malaria in Myanmar (where the disease burden is higher than in Thailand), frequent population movement, and differences in adequate control measures on the two sides of the border. In this study the authors evaluated malaria prevalence and incidence in the mobile population on the Myanmar side of the border between 1 October 1999 and 30 September 2011 to assess whether increasing access to early diagnosis and treatment with ACT was associated with a decline in the malaria burden.

**What Did the Researchers Do and Find?:**

The Shoklo Malaria Research Unit (SMRU) has been working on the Thai–Myanmar border for 25 years providing early diagnosis and treatment of malaria and has extended its services from two to 11 health care facilities (health posts) on the Myanmar side of the border over the past few years. In order to evaluate any changes in the malaria burden since the expansion of services, the researchers recorded the number of consultations in all SMRU clinics and health posts with confirmed malaria diagnosis and tracked changes in the prevalence of malaria in the population on the Myanmar side of the border (via cross-sectional surveys in villages). The researchers also assessed the incidence of malaria in a cohort of pregnant women living on both sides of the border and monitored antimalarial drug efficacy over this time period.

The researchers found that although the mobile population on the Thai side of the border remained constant, the population in villages covered by the clinics and health posts in the border area increased four-fold. Over the time period, the researchers found that the number of confirmed malaria cases (*P. falciparum*) increased initially, rising from just over 5,000 in 2000 to a peak of 13,764 in 2006, and then declined to just over 3,500 in 2011. A striking finding was the predominance of infections in young adult males (50,316/90,321; 55.7%). Encouragingly, the percentage of consultations due to malaria in children under five years fell from 78% to 7%, and the incidence of malaria declined from 1.1 to 0.1 episodes per pregnant woman-year. In addition, the proportion of patients admitted to hospital with severe disease was stable, and the number of deaths from malaria remained extremely low, with an overall case fatality rate of 0.05%. The researchers also found that the ratio of *P. falciparum* to *P. vivax* infections declined from 1.4 to 0.7, and the prevalence of *P. falciparum* decreased from 24.3% to 3.4%. However, worryingly, in the small number of patients undertaking drug efficacy tests, the drug efficacy of artesunate declined steadily, with the proportion of patients still infected with malaria at day 3 of treatment increasing from 0% in 2000 to 28% in 2011.

**What Do These Findings Mean?:**

These findings indicate that despite the emergence of resistance to artesunate in *P. falciparum*, and the decline in the efficacy of ACT, the strategy of early diagnosis and treatment with ACTs has been associated with a reduction in malaria in the population living on the Thai–Myanmar border. Furthermore, these findings suggest that an aggressive strategy based on early detection and treatment of cases, combined with vector control and information, could be the way forward to eliminate malaria. Although there were only a small number of patients involved in drug efficacy tests in 2011, this study shows that alternative fixed combination treatments are needed urgently to replace the failing first-line regimen of mefloquine and artesunate.

**Additional Information:**

Please access these websites via the online version of this summary at http://dx.doi.org/10.1371/journal.pmed.1001398.

- More information about the Shoklo Malaria Research Unit is available
- The World Health Organization website has more information about antimalarial drug efficacy and drug resistance
- The Bill & Melinda Gates Foundation website tells the malaria resistance story

## Introduction

The past decade has seen encouraging progress in the control of malaria worldwide. According to the World Health Organization's *World Malaria Report 2011* [1], several countries in sub-Saharan Africa have registered very significant decreases in the number of *Plasmodium falciparum* malaria cases. In other continents, malaria has receded too, particularly in Southeast Asia, although the precise number of cases in some countries of the subcontinent may have been grossly underestimated [2]. Economic development, urbanisation, and unprecedented financial support have all contributed to these successes against malaria. Renewed efforts in vector control, insecticide-treated net distribution, and the deployment of artemisinin-based combination treatments (ACTs) have also contributed to the reduction of morbidity and mortality associated with *P. falciparum*, and this trend is particularly prominent in areas of low transmission such as in Southeast Asia.

These trends have boosted efforts towards malaria elimination. However, these gains are now threatened by the emergence in Southeast Asia of *P. falciparum* isolates that exhibit resistance to artesunate [3]. This resistance is characterised by a slow parasite clearance rate observed in patients treated with artesunate, but the precise mechanism remains unknown. Whether or not this particular form of resistance has spread beyond Southeast Asia is currently uncertain, but confirmed resistance surrounding the Thai–Myanmar border is attributable to changes in the parasite's genetic make-up [4]. It is not known whether the resistant parasites on the Thai–Myanmar border are related to those in Cambodia or whether resistance to artemisinin has emerged independently, but there is considerable concern that if this resistance to artemisinin spreads or emerges elsewhere because of widespread use, it will seriously compromise malaria control programs and threaten the lives of millions worldwide. During the previous decades, the spread of chloroquine- and sulphadoxine-pyrimethamine-resistant parasites from Southeast Asia to Africa resulted in a dramatic rise in mortality from increased perennial or epidemic transmission [5],[6]. Resistance to artemisinin could trigger a catastrophic resurgence in malaria in many parts of the world and compromise the progress made in the treatment of severe malaria [7],[8]. With substantial international support, containment efforts have started in Cambodia and bordering Thailand. The strategy is based on active detection of parasite carriers using molecular tools, but the impact is difficult to measure in the absence of a molecular marker of artemisinin resistance.

The largest focus of *P. falciparum* malaria in this region is situated in Myanmar, with a reported annual caseload of 70,941 in 2010 [1]. It is therefore essential to suppress malaria transmission as much as possible on the border between Myanmar and Thailand to avoid the spread of resistance to artemisinin to neighbouring countries and beyond. Between 1994 and 1999 we observed the impact of early detection and treatment (EDT) with ACT in populations of refugees living on the Thai–Myanmar border [9]. Ten years later we reported a similar impact in a larger population living in the region, including villagers, migrant workers, and refugees [10]. In the meantime, as per national policies, Thailand (in 1995), Vietnam (in 1995), Cambodia (in 2000), and Myanmar (in 2002) all adopted ACT as first-line therapy for *P. falciparum* infections. Malaria control in border areas is particularly challenging, especially if effective control measures are not deployed on both sides of the border. The combination of a constant reservoir of malaria in Myanmar, where the disease burden is higher than in Thailand, and frequent population movement mean that providing control measures based on EDT alone to migrants on the Myanmar side of the border might be expected to have a lower impact on local malaria transmission and incidence than in a more enclosed population, such as in large refugee camps or in villages on the Thai side of the border, where similar measures have been highly successful.

We evaluated malaria prevalence and incidence in the mobile and migrant population on the Myanmar side of the border between 1 October 1999 and 30 September 2011 to assess whether increasing access to EDT with ACT for this population was associated with a decline in the malaria burden.

## Methods

### Setting

The border between Thailand and Myanmar (Burma) is 2,107 km long and is mostly forested and mountainous. It is inhabited by a mosaic of ethnic groups and is characterised by intense migration fluxes between the two countries. Decades of internal conflicts in Myanmar have resulted in massive population displacements, and over 150,000 refugees now live in camps in Thailand. Economic stagnation has also prompted the migration of millions of people to Thailand in search of work, especially since the mid-1990s. This region is endemic for malaria (all four types, namely, *P. falciparum*, *P. vivax*, *P. ovale*, and *P. malariae*), and *P. falciparum* is highly drug resistant. The migration of gem miners from western Cambodia to Myanmar in the 1960s is thought to have played a major role in the spread of resistance to chloroquine [11]. The main vectors of malaria are the mosquitoes *Anopheles minimus*, *A. dirus*, and *A. maculatus*, and the transmission is low but unstable. The population has very little naturally acquired immunity against malaria, and all age groups are affected. In 1985, malaria was the main cause of consultations and mortality in the Karen refugee camps [12].

### Population

**Figure 1:**
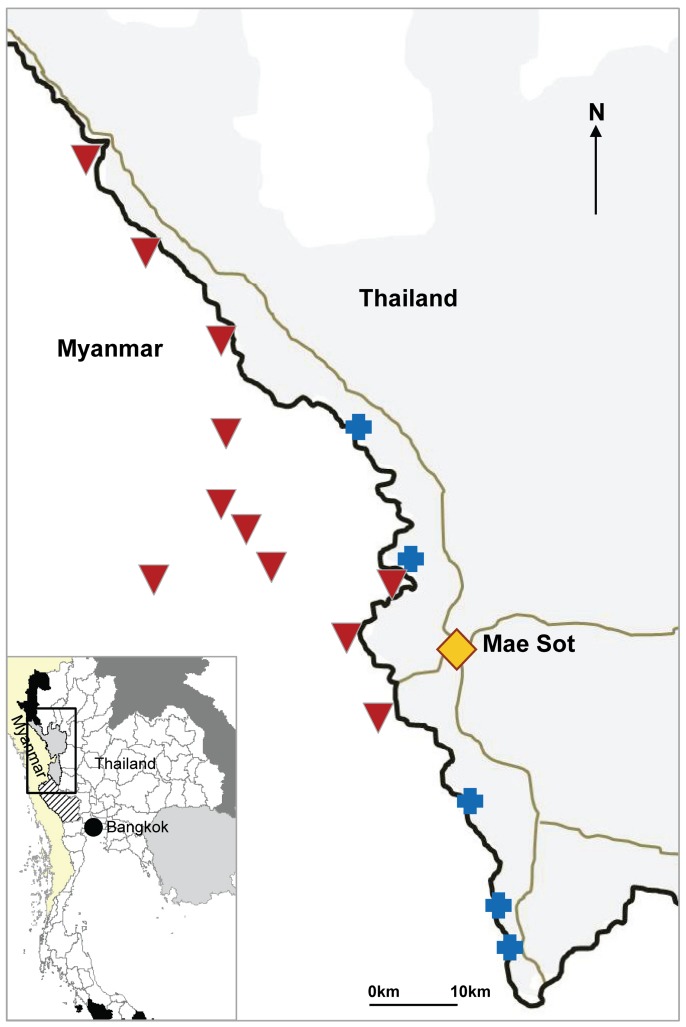
Location of SMRU clinics and cross-border health posts run by village health workers. Blue crosses indicate SMRU clinics; inverted red triangles indicate SMRU health posts. The Thai–Myanmar border is represented by a black line, and the main roads by grey lines.

The catchment area of the five Shoklo Malaria Research Unit (SMRU) clinics extends over 120 km of the border (see Figure 1). The population living along the border is diverse and is composed of three main groups: a relatively stable population living in established villages inside Myanmar for a number of years; a much more mobile group, mainly adult workers migrating according to work availability; and newly arrived people either displaced or in search of employment.

Based on village population lists, the population living in villages on the Myanmar side has been estimated to be 42,000 people, with numbers updated during malariometric surveys and bed net distributions. This population size has grown steadily through the years. The ratio of men to women is estimated at 1, and ∼40% of the population is under 15 y of age, a population structure similar to that of the refugee camps. Villages in the catchment area are mainly located along the Moei river (the border between Myanmar and Thailand) but occasionally are as far as 30–40 km inside Myanmar.

The size of the mobile population is more difficult to assess; in 2004, the Thai Ministry of Interior estimated the number of registered migrants living in Tak province at ∼125,000. Another 50,000–100,000 migrants are thought to be living along the border without registration (data from the Tak public health services). The majority of those migrants are living in the three districts where SMRU clinics are located (namely, Mae Ramat, Mae Sot, and Phop Phra districts). In 2010, the Tak Industrial Federation estimated that 154,000 to 190,000 migrants (both registered and unregistered) were living in Tak province.

### Expanding Cross-Border Access to EDT

Access to malaria diagnosis and treatment on the Myanmar side of the border is poor, because of travel limitations and insecurity. Health care is provided by local organisations, and coverage is limited. In general, patients with more severe illness seek treatment in Thailand. From 1999 to 2005, two “health posts” were run by locally selected health workers; they were trained by the SMRU staff to use rapid diagnostic tests (RDTs) and microscopy and to provide appropriate treatment following the protocols used in the SMRU clinics. After 2008, nine health posts were established on the Myanmar side but without microscopy. (see Figure 1). The health posts are operated and supported as follows. Severe cases are referred to the nearest medical facility. Antimalarial drugs (artemisinin-based combinations as well as chloroquine for the treatment of non-falciparum malaria) and antipyretics are supplied on a regular basis to avoid shortages or stock-outs. Supervision of the work is done routinely, and quality control (QC) of malaria diagnosis is performed at the SMRU laboratory in Mae Sot. Pregnant women seeking care at the health posts are encouraged to attend antenatal care at the nearest SMRU clinic on the Thai side. Insecticide-impregnated bed nets are distributed to all patients with confirmed malaria seen in SMRU clinics, as per the requirements of the clinic's funding agencies.

### Study Design

In order to evaluate changes in the malaria burden since access to EDT was expanded, we recorded the number of consultations in all clinics with confirmed malaria diagnosis, changes in the prevalence of malaria in the populations on the Myanmar side of the border by means of cross-sectional surveys, and the incidence of malaria in a cohort of pregnant women living on both sides of the border but followed in clinics on the Thai side. Other factors that can affect malaria incidence were monitored, i.e., in vivo and in vitro antimalarial drug efficacy, bed net distribution, and climatic conditions. The observation period extended from 1 October 1999 to 30 September 2011.

### Monitoring the Number of Malaria Cases

In the cross-border health posts (Myanmar side), malaria cases were detected by village health workers. Data were obtained weekly, and compiled on site by age group and malaria species. No gender distribution was available. All cases of *P. falciparum* reported were confirmed by either RDT (nine health posts) or microscopy (two health posts).

For each individual malaria case detected in the SMRU clinics (Thai side), demographic characteristics and type of malaria test and its result were recorded. Species were confirmed systematically by slide microscopy only for children under 5 y and pregnant women because some of the RDTs used can only detect *P. falciparum*. However, one week each month, all patients presenting at the SMRU clinics with malaria symptoms were tested by blood smear microscopy, allowing the calculation of changes in the *P. falciparum*/*P. vivax* ratio over time. The presence of gametocytes was reported routinely for each malaria smear done in the SMRU clinics. Hospitalisations due to severe malaria have been systematically recorded since 2003.

QC of the malaria diagnostic tests was implemented at all sites. All RDTs performed in the field by village health workers, mostly Paracheck-Pf, an HRP-II-based RDT detecting only *P. falciparum*, and Optimal-IT, a pLDH-based test detecting falciparum and non-falciparum species, were retained in zip-sealed plastic bags on site and reread later by a senior technician in Mae Sot. Although, this QC method has limitations, as RDT results do not always remain stable over time, it enables a check on the accuracy of data recording and can detect major errors in test result interpretation in the field that warrant further investigation or refresher training.

For SMRU clinics and health posts with a field laboratory performing microscopy, a standardised protocol for internal QC has been in place since 1997. Briefly, each field laboratory stores all slides in zip-sealed plastic bags, separating positive from negative slides. These are sampled several times a year and sent to the central laboratory in Mae Sot for cross-checking and quality assessment of the sensitivity, specificity, and negative and positive predictive values of the tests. Any discrepancies between results required a third and final reading by the head of the laboratory department.

### Malaria Incidence: Follow-Up of the Cohort of Pregnant Women

Pregnant women attended weekly antenatal care clinics until delivery. In addition to routine antenatal care, a malaria smear was done at each consultation, whether the woman was symptomatic or not, and her haematocrit was measured every fortnight. Pregnant women were also encouraged to seek care immediately in case of fever or symptoms suggestive of malaria. Women found to be parasitaemic were treated for malaria, regardless of their symptoms.

### Malariometric Cross-Sectional Surveys

Cross-sectional surveys in villages and migrant communities were conducted annually in order to detect changes in the transmission of *P. falciparum* and *P. vivax*. We surveyed one or two villages each year between 2000 and 2003; in 2006–2008, prior to expanding malaria control on the Myanmar side, we surveyed up to five villages annually. Most of the surveys were done during the rainy season, when malaria is at its peak. Exhaustive population surveys were done in villages with fewer than 500 inhabitants; for larger villages, 25% of the houses were randomly selected, and all residents in those houses were screened. Participation to the survey was voluntary. Each participant was given a unique identifier, was asked basic demographic information, and had a malaria smear performed. Anyone presenting with fever or a history of fever in the previous days also underwent an RDT for malaria, and if the test was positive, the individual was immediately treated. Malaria smears were considered negative if the technician did not find any parasites in 100 oil-immersion fields (1,000×) on a thick blood film. Results of the malaria smear screening were obtained within 24–48 h, and all people with a positive malaria smear result were treated.

### In Vivo Efficacy of Mefloquine-Artesunate

We monitored the efficacy of mefloquine (8 mg/kg/d) and artesunate (4 mg/kg/d) over 3 d for the treatment of patients with uncomplicated *P. falciparum* malaria confirmed microscopically annually. After giving written consent, patients were followed daily until resolution of their parasitaemia and then weekly for 6 wk. Recrudescence between days 5 and 42 was differentiated from a new infection using parasite genotyping by PCR [13]. Patients with indeterminate genotyping results and new infections were censored at the date of the reappearance of *P. falciparum* parasites. Delayed parasite clearance was considered to be present if patients were still parasitaemic 72 h (day 3) after the baseline positive malaria smear.

### In Vitro Antimalarial Drug Studies

Parasite isolates were obtained from non-pregnant patients with uncomplicated acute *P. falciparum* malaria primary infection attending the SMRU clinics if they had at least five parasites per 1,000 red blood cells and consented to give 5 ml of blood. Drug susceptibility was tested using the hypoxanthine uptake method. The reproducibility of IC_50_ measurements was assessed using cloned K1 isolates of *P. falciparum*.

### Climate

Monthly climatic conditions (rainfall and mean temperature) were obtained from Mae Sot (central area) and Umphang (southern area) meteorological stations and from the Tha Song Yang health department (northern area). Annual trends were obtained by averaging monthly rainfall (in millimetres) and mean temperature (in degrees Celsius) from the different meteorological stations.

### Statistical Analysis

Annual incidence rates in pregnancy for *P. falciparum* and for *P. vivax* were calculated separately. Only the first positive malaria smear of each species was considered and counted to calculate the number of new malaria episodes per pregnant woman. Women with negative malaria smears throughout their pregnancy were considered free of malaria; the period between the first antenatal care visit and the pregnancy outcome was considered the period “at risk” and was measured in weeks. In addition to incidence, the annual cumulative proportion of women who experienced at least one malaria episode during their pregnancy was calculated for both *P. falciparum* and *P. vivax*. Anaemia was reported as present if the haematocrit was <30% at least once during the pregnancy, and is presented as a proportion of the total number of women per year.

Proportions of positive cases found during malariometric surveys were stratified by plasmodial species, age, and gender, and were compared by chi-square test with Bonferroni correction.

Mefloquine-artesunate (MAS_3_) PCR-adjusted cure rates were estimated using Kaplan-Meier survival analysis. Patients who did not complete the 3-d treatment course or did not have at least 3 d of follow-up were excluded from the parasite clearance time analysis.

In vitro antimalarial dose–response curves were measured by a nonlinear regression function to determine the IC_50_ (expressed in nanograms/millilitre) using WinNonlin (version 4.1, Pharsight Corporation), and data were analysed using SPSS for Windows (version 15, SPSS). Temporal trends in geometric mean IC_50_ values of mefloquine, dihydroartemisinin, and artesunate were analysed (ANOVA method).

## Results

### Population

The changes in the estimated population figures are shown in Table 1. Although these are only estimates, they indicate that the mobile population living on the Thai side has remained stable (approximately 200,000), while the population living in established villages in the area covered by SMRU clinics and the health posts (including the settlements on the Myanmar side) has increased 4-fold.

**Table 1:** Population estimates between 2000 and 2011.

Population	Year		
	2000	2004	2011
Migrants on Thai side (registered and unregistered)	150,000–200,000	175,000–225,000	154,000–190,000
Villagers in Myanmar living within the SMRU catchment area	8,000–10,000	10,000–15,000	30,000–45,000

### Number of *P. falciparum* Infections

**Figure 2:**
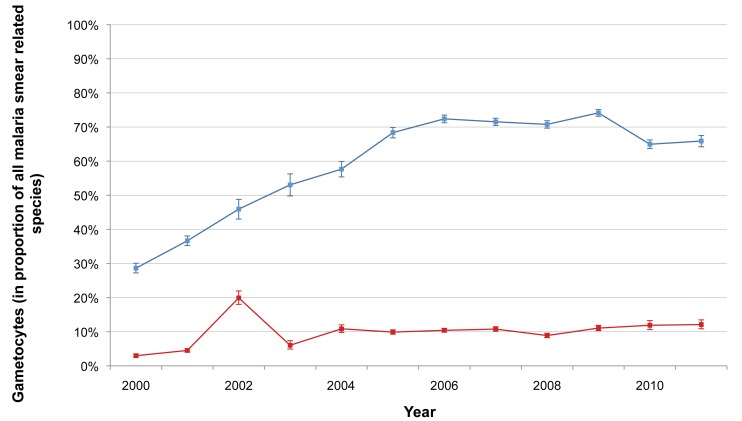
Proportion of malaria smears with *P. falciparum* or *P. vivax* gametocytes. Annual proportion of all patients whose smears were positive for *P. falciparum* malaria who had *P. falciparum* gametocytes present on their malaria smear (red), and those whose smears were positive for *P. vivax* malaria who had *P. vivax* gametocytes present on their malaria smear (blue). Bars indicate 95% CIs.

The total number of *P. falciparum* infections we detected per year in SMRU clinics and in the village health posts is shown in Table 2. The number of cases detected increased initially with the opening of new facilities but thereafter gradually decreased, while the overall number of consultations (malaria-related or not) steadily increased from 25,345 to 69,000, reflecting the rise of the general population living in the area. Table 3 shows the distribution by age and gender of the *P. falciparum* cases detected in the SMRU clinics. The most striking finding is the predominance of infections (50,316/90,321; 55.7% [95% CI 55.4–56.0]) in young adult males, probably reflecting a higher exposure because of their occupations. The proportion of patients who presented with gametocytes increased significantly from 3.0% (95% CI 2.3–3.5) (149/5,010) in 2000 to 12.1% (95% CI 10.9–13.5) (296/2,446) in 2011 (chi-square test for trend, *p<*0.001) (Figure 2).

**Table 2:** Annual total number of consultations and number of *P. falciparum* infections detected in SMRU clinics and health posts.

Year	Number of Health Facilities	Total PF in SMRU Clinics	Total Consultations in SMRU Clinics	Total PF in Health Posts	Total Consultations in Health Posts	Total PF	Total Consultations
2000	3	5,035	22,840	865	2,505	5,900	25,345
2001	4	7,102	21,197	2,195	6,287	9,297	27,484
2002	4	4,098	21,796	2,334	9,254	6,432	31,050
2003	4	4,249	19,035	2,267	6,956	6,516	25,991
2004	5	4,015	23,410	1,728	4,879	5,743	28,289
2005	6	9,117	38,044	1,596	4,500	10,713	42,544
2006	6	13,764	54,488	1,840	5,519	15,604	60,007
2007	6	12,290	59,804	1,203	4,852	13,493	64,656
2008	9	13,201	62,340	4,324	13,673	17,525	76,013
2009	12	9,795	61,527	6,855	25,839	16,650	87,366
2010	15	4,681	55,281	4,487	21,302	9,168	76,583
2011	16	3,570	48,392	5,552	20,608	9,122	69,000

PF, *P. falciparum* infections.

**Table 3:** Total *P. falciparum* malaria cases confirmed and treated in SMRU clinics by sex and age group (and as percentage of total annual number of *P. falciparum* cases).

Year	Age						Total *P.falciparum* Cases	Overall Male: Female Ratio
	<5 y		5–15 y		>15 y			
	Male	Female	Male	Female	Male	Female		
2000	210 (4%)	213 (4%)	653 (13%)	491 (10%)	2,669 (53%)	788 (16%)	5,024	2.4
2001	259 (4%)	238 (3%)	984 (14%)	656 (9%)	3,825 (54%)	1,137 (16%)	7,099	2.5
2002	174 (4%)	162 (4%)	591 (14%)	418 (10%)	2,105 (51%)	644 (16%)	4,094	2.3
2003	133 (4%)	136 (4%)	481 (13%)	321 (9%)	2,057 (56%)	552 (15%)	3,680	2.6
2004	168 (4%)	145 (4%)	538 (13%)	389 (10%)	2,061 (51%)	714 (18%)	4,015	2.2
2005	327 (4%)	256 (3%)	1,171 (13%)	935 (10%)	4,813 (53%)	1,611 (18%)	9,113	2.3
2006	485 (4%)	398 (3%)	1,787 (13%)	1,220 (9%)	7,749 (56%)	2,125 (15%)	13,764	2.7
2007	382 (3%)	285 (2%)	1,719 (14%)	1,087 (9%)	6,955 (57%)	1,862 (15%)	12,290	2.8
2008	344 (3%)	325 (2%)	1,736 (13%)	1,189 (9%)	7,538 (57%)	2,067 (16%)	13,199	2.7
2009	266 (3%)	234 (2%)	1,282 (13%)	828 (8%)	5,685 (58%)	1,500 (15%)	9,795	2.8
2010	90 (2%)	92 (2%)	573(12%)	347 (7%)	2,836 (61%)	741 (16%)	4,679	3.0
2011	117 (3%)	86 (2%)	510 (14%)	273 (8%)	2,023 (57%)	560 (16%)	3,569	2.9

Total annual numbers of *P. falciparum* cases reported in this table are smaller than those reported in Table 2 because of some missing data on either age or sex.

### Number of *P. vivax* Infections

The annual numbers of *P. vivax* (and *P. falciparum*) infections in children under 5 y that presented at the SMRU clinics are shown in Table 4. The percentage of consultations due to malaria decreased over time (from 1,048/1,344, 78% [95% CI 76–80], to 767/11,542, 7% [95% CI 6.2–7.1]; chi-square test for trend, *p<*0.001).There was a significant decrease in the ratio of *P. falciparum* infections to *P. vivax* infections in the children during the study period, from 0.7 (95% CI 0.6–0.7) to 0.3 (95% CI 0.2–0.3) (chi-square test for trend, *p<*0.001). This finding was also evident (in all age groups) in the data compiled during the weeks when all patients presenting at SMRU clinics with malaria symptoms had a malaria smear, as shown in Table 5. The largest change was in the adult population, in which the ratio of *P. falciparum* infections to *P. vivax* infections decreased from 1.8 (95% CI 1.7–1.9) to 0.8 (95% CI 0.8–0.8) (chi-square test for trend, *p<*0.001). The proportion of patients who presented with *P. vivax* gametocytes steadily increased over the years to a plateau in 2006 (difference in proportion between 2000 and 2006 43.8% [95% CI 41.9–45.6]; chi-square test, *p<*0.001) (Figure 2).

**Table 4:** Total *P. falciparum* and *P. vivax* malaria cases confirmed by malaria smear among all children under 5 y old treated in SMRU clinics.

Year	All Consultations <5 y	Number of *P. falciparum* Cases (Percent of Consultations)	Number of *P. vivax* Cases (Percent of Consultations)	Ratio PF:PV
2000	1,344	418 (31%)	630 (47%)	0.7
2001	3,205	441 (14%)	691 (22%)	0.6
2002	3,182	137 (4%)	213 (7%)	0.6
2003	3,756	169 (4%)	239 (6%)	0.7
2004	5,902	280 (5%)	509 (9%)	0.6
2005	9,617	578 (6%)	946 (10%)	0.6
2006	12,032	871 (7%)	1,638 (14%)	0.5
2007	13,641	659 (5%)	1,707 (13%)	0.4
2008	12,700	652 (5%)	1,588 (13%)	0.4
2009	12,062	487 (4%)	1,643 (14%)	0.3
2010	12,747	133 (1%)	788 (6%)	0.2
2011	11,542	161 (1%)	606 (5%)	0.3

Ratio PF:PV is the ratio of *P. falciparum* malaria cases to *P. vivax* malaria cases; a ratio above 1 indicates a predominance of *P. falciparum*, and a ratio below 1 a predominance of *P. vivax*.

**Table 5:** Ratio of *P. falciparum* to *P. vivax* malaria cases confirmed during weeks when all patients presenting to SMRU clinics with malaria symptoms had a malaria smear, by age group.

Year	Age			Overall
	<5 y	5–15 y	>15 y	
2000	0.7	1.0	1.8	1.4
2001	0.7	1.2	1.7	1.7
2002	0.1	0.4	1.1	0.7
2003	0.4	0.5	1.0	0.8
2004	0.5	0.8	1.5	1.1
2005	0.6	1.3	2.1	1.6
2006	0.5	1.0	1.5	1.2
2007	0.4	0.7	1.1	0.9
2008	0.3	0.6	1.1	0.8
2009	0.3	0.4	0.7	0.6
2010	0.1	0.2	0.4	0.3
2011	0.5	0.7	0.8	0.7

One week each month, all patients presenting at the SMRU clinics with malaria symptoms were tested by microscopy, allowing the calculation of the ratio of *P. falciparum* to *P. vivax* infection; a ratio above 1 indicates a predominance of *P. falciparum*, and a ratio below 1 a predominance of *P. vivax*.

### Hospitalised Cases and Mortality

Table 6 shows the number of patients hospitalised for malaria during the study period and the case fatality rate. The proportion of patients hospitalised for either uncomplicated hyperparasitaemia or severe disease was stable, 4.0% (95% CI 3.9–4.2) (3,022 hospitalisations/75,126 total patients with falciparum malaria). The number of deaths remained very low: the overall case fatality rate was 0.05% (95% CI 0.04–0.07), ranging across years from 0.01% (95% CI 0.00–0.06) to 0.11% (95% CI 0.05–0.25). Overall, only two patients (0.003% [95% CI 0.000–0.010] of 72,104 patients treated in ambulatory care in the outpatient department) died after receiving an oral artemisinin-based treatment for an uncomplicated *P. falciparum* infection. Forty-eight patients were hospitalised with a *P. vivax* infection and a concomitant condition between 2007 and 2011, but none were fatal or associated with signs of severe malaria.

**Table 6:** Hyperparasitaemia and severe malaria cases hospitalised in SMRU clinics, and case fatality rate among non-pregnant patients, from 2003 to 2011.

Year	OPD+IPD Malaria: Total PF	OPD Malaria: Total PF	IPD Malaria			Malaria Deaths	Case Fatality Rate
			Total PF^a^	PF Hyperparasitaemia^b^	Severe PF Malaria^b^		
2003	4,235	4,061	174 (4.1%)	115 (66%)	59 (34%)	1	0.02%
2004	3,945	3,760	185 (4.7%)	104 (56%)	81 (44%)	1	0.03%
2005	8,937	8,752	185 (2.1%)	113 (61%)	72 (39%)	6	0.07%
2006	13,648	13,306	342 (2.5%)	200 (58%)	142 (42%)	11	0.08%
2007	12,599	12,013	586 (4.7%)	469 (80%)	117 (20%)	5	0.04%
2008	13,412	12,836	576 (4.3%)	416 (72%)	160 (28%)	7	0.05%
2009	10,117	9,553	564 (5.6%)	380 (67%)	184 (33%)	1	0.01%
2010	4,746	4,514	232 (4.9%)	151 (65%)	81 (35%)	5	0.11%
2011	3,487	3,309	178 (5.1%)	140 (79%)	38 (21%)	2	0.06%

a Percentages in parentheses indicate the percent of all malaria cases treated in the inpatient department for a given year.

b Percentages in parentheses indicate the percent of each category among those treated in the inpatient department for a given year.

IPD, hospitalisation in inpatient department; OPD, ambulatory care in outpatient department, PF, *P. falciparum* malaria.

### Laboratory Quality Control

Between 1 January 2000 and 31 December 2011 a total of 42,806 malaria smears were rechecked. This figure corresponds to an average of 3.6 unannounced rounds of QC per site per year. The mean sensitivity, specificity, positive predictive value, negative predictive value, and Kappa value for species differentiation were 95.7%, 99.8%, 99.7%, 98.0%, and 0.95, respectively. Out of 14,426 Paracheck-Pf tests that could be verified, 2.3% (95% CI 2.1–2.6) (335/14,426) had an interpretation error. Out of 9,569 Optimal-IT tests verified, 1.1% (95% CI 1.0–1.4) (109/9,569) were wrongly interpreted. However, delays in double-checking the RDTs may have affected the reliability of these results.

### Malaria in Pregnancy

**Figure 3:**
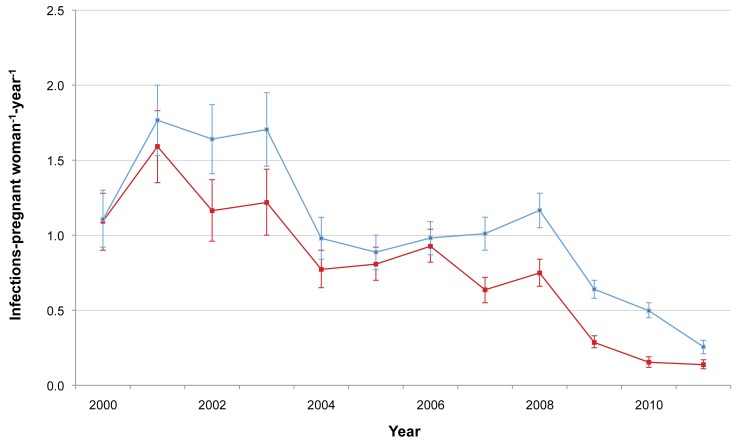
*P. falciparum* and *P. vivax* malaria incidence among pregnant women. Red line indicates *P. falciparum;* blue line indicates *P. vivax*. Bars indicate 95% CIs.

**Figure 4:**
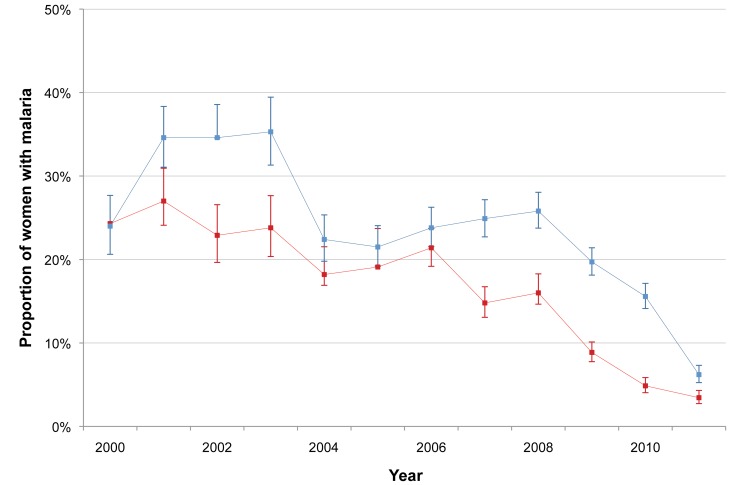
Annual proportion of *P. falciparum* and *P. vivax* infections among pregnant women. Red line indicates *P. falciparum;* blue line indicates *P. vivax*. Bars indicate 95% CIs.

**Figure 5:**
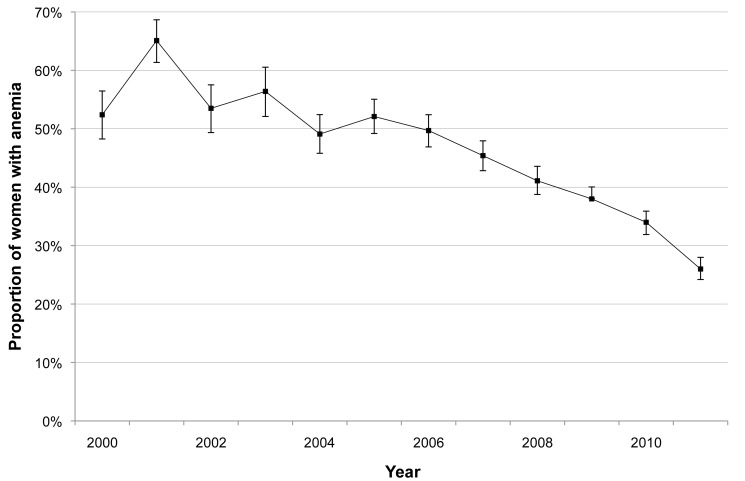
Proportion of pregnant women with anaemia during their pregnancy. Anaemia was considered present if there was at least one haematocrit value<30% during the course of the pregnancy. Bars indicate 95% CIs.

The incidence of *P. falciparum* and *P. vivax* infection in pregnant women is shown in Figure 3. There was a marked decline in the incidence of *P. falciparum*, from 1.09 to 0.14 infections·woman^−1^·year^−1^ (Student's *t* test, *p<*0.001), while *P. vivax* incidence declined only after 2003 (from 1.70 to 0.26 infections·woman^−1^·year^−1^; Student's *t* test, *p<*0.001). The cumulative proportion of women infected during pregnancy is illustrated in Figure 4 and clearly shows the decline in malaria exposure in this group from 24.3% (95% CI 21.0–28.0) (143/588) to 3.4% (95% CI 2.8–4.3) (76/2,207) for *P. falciparum* and from 24.0% (95% CI 20.7–27.6) (141/588) to 6.2% (95% CI 5.3–7.3) (137/2,207) for *P. vivax*, during the 12-y period. During the same period, the proportion of pregnant women with at least one haematocrit measurement below 30% also declined significantly (from 52.4% [95% CI 48.3–56.5] to 26.0% [95% CI 24.2–28.0]; chi-square test for trend, *p<*0.001) (Figure 5).

### Cross-Sectional Malariometric Surveys

**Figure 6:**
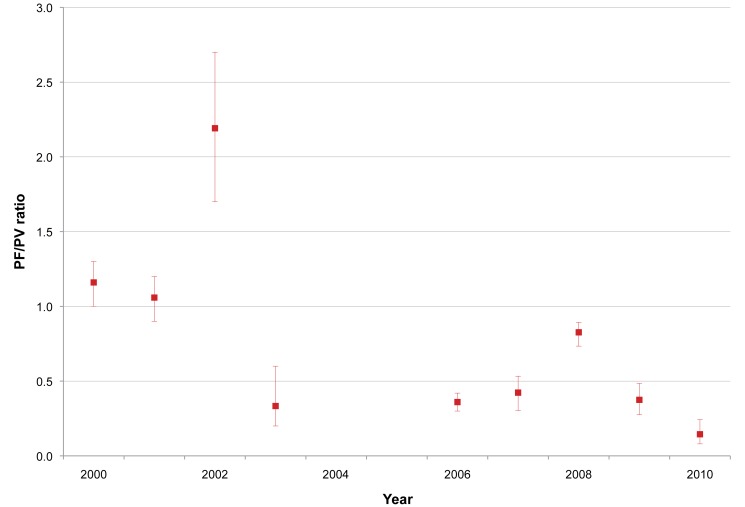
*P. falciparum* infection/*P. vivax* infection ratio from village surveys in Myanmar within SMRU clinic/health post catchment area. Red squares indicate the *P. falciparum* infection/*P. vivax* infection (PF/PV) ratio; bars indicate 95% CIs. A ratio above 1 indicates the predominance of *P. falciparum* over *P. vivax*; a ratio below 1 indicates the predominance of *P. vivax*.

We conducted 24 cross-sectional surveys during the study period in 8,106 people from communities and villages on the Myanmar side of the border. The results are summarised in Table 7, and the ratio of *P. falciparum* to *P. vivax* infection is shown in Figure 6. *P. falciparum* was the predominant species during surveys conducted in Myanmar villages prior to 2003, with an annual prevalence ranging from 8.0% (95% CI 5.7–11.3) (29/361) to 24.6% (95% CI 20.7–28.9) (103/419). In 2006, *P. falciparum* prevalence decreased by 2/3 and continued to decrease until 2010, when 12/900 (1.3% [95% CI 0.8–2.3]) persons tested positive for *P. falciparum*. In contrast, *P. vivax* prevalence remained relatively constant over the years, with an averaged prevalence of 8.9% (95% CI 8.4–9.6) (728/8,106), and as of 2010 was the predominant species along the border area. As already observed among patients with confirmed malaria seeking care at the SMRU clinics, there was a predominance of *P. falciparum* infections in young adult males (41% [95% CI 36–46] of all *P. falciparum* infections found). *P. vivax*, by contrast, was predominant in the age group 5–15 y (12.8% [95% CI 11.6–14.2] [315/2,457] versus 6.5% [95% CI 5.4–7.9] [100/1,530] in age group <5 y and 7.6% (95% CI 6.8–8.5) (313/4,119) in age group >15 y; chi-square test, *p<*0.001).

**Table 7:** Prevalence of *P. falciparum* and *P. vivax* infection and of gametocyte carriage during surveys of Myanmar villages within SMRU clinics and health posts catchment area.

Year	Population Screened	*P. falciparum*	*P. vivax*	PFG	PVG
2000	361	8.0% (5.7–11.3)	6.9% (4.7–10.0)	3.3% (1.9–5.7)	1.1% (0.4–2.8)
2001	99	18.2% (11.8–26.9)	17.2% (11.0–25.8)	0% (0.0–3.7)	10.1% (5.6–17.6)
2002	419	24.6% (20.7–28.9)	11.2% (8.5–14.6)	8.8% (6.5–11.9)	4.1% (2.6–6.4)
2003	284	2.8% (1.4–5.5)	8.5% (5.7–12.3)	0.4% (0.0–2.0)	0.7% (0.2–2.5)
2006	2,755	3.4% (2.8–4.2)	9.7% (8.7–10.9)	1.1% (0.8–1.6)	3.2% (2.6–3.9)
2007	966	3.4% (2.4–4.8)	8.1% (6.5–10.0)	0.9% (0.5–1.8)	2.6% (1.8–3.8)
2008	1,158	7.0% (5.7–8.6)	8.5% (7.0–10.2)	2.3% (1.6–3.4)	2.3% (1.6–3.4)
2009	1,164	2.8% (2.0–4.0)	7.6% (6.2–9.2)	1.3% (0.8–2.1)	2.7% (1.9–3.8)
2010	900	1.3% (0.8–2.3)	9.2% (7.5–11.3)	0.4% (0.2–1.1)	2.1% (1.4–3.3)

95% CIs given in parentheses. No surveys with malaria smear were done in 2004, 2005, and 2011.

PFG, *P. falciparum* gametocytes; PVG, *P. vivax* gametocytes.

### In Vivo Efficacy of Mefloquine-Artesunate

**Figure 7:**
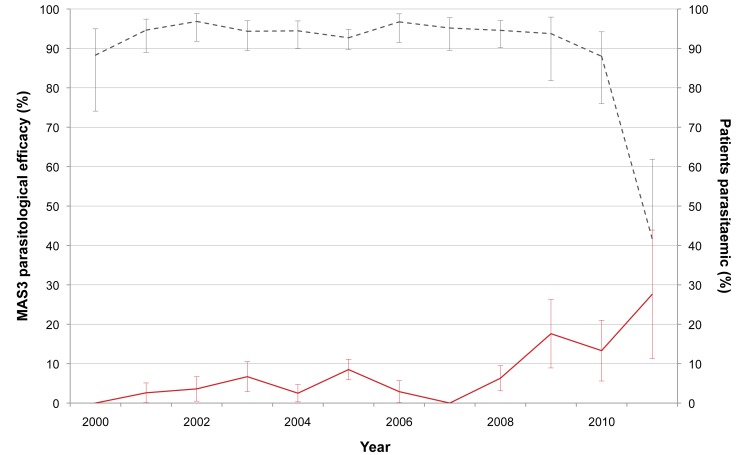
Day-42 PCR-adjusted MAS_3_ parasitological efficacy and proportion of patients still parasitaemic at day 3. This graph shows the changes in day-42 PCR-adjusted MAS_3_ parasitological efficacy (black dashed line) and proportion of patients still parasitaemic at day 3 (red line) from 2000 to 2011. Bars indicate 95% CIs.

We enrolled 1,926 people in studies to determine the efficacy of MAS_3_ between 1 October 1999 and 30 September 2011; 75/1,926 people (3.9%) did not complete the 3-d treatment course or did not have at least 3 d of follow-up and were excluded from the parasite clearance time analysis, but they remained included for the cure rate analysis. The day-42 PCR-corrected cure rates and the proportion of patients still parasitaemic at day 3 are shown in Figure 7. There was a significant decline in the efficacy of MAS_3_ over the years (hazard ratio = 1.12 [95% CI 1.04–1.21], *p<*0.001) and a significant lengthening of the parasite clearance time (none of the 46 patients had a delayed [>3 d] clearance of parasitaemia in 2000, but 8 of 29 [28% (95% CI 15–46)] did in 2011; chi-square test for trend, *p* = 0.003).

### In Vitro Sensitivity

**Figure 8:**
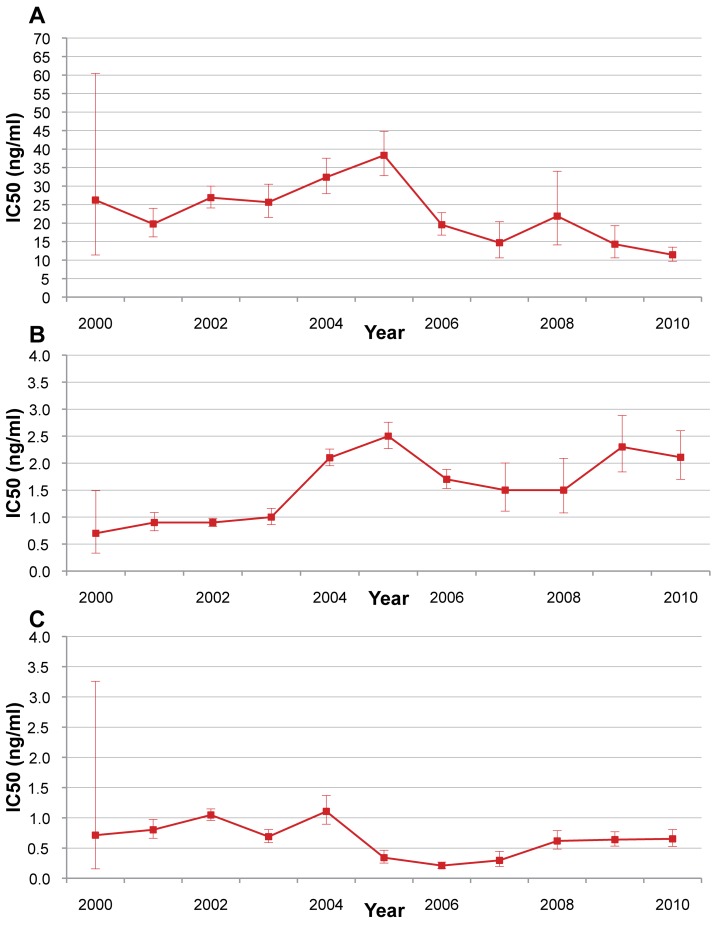
*P. falciparum* isolate in vitro sensitivity to mefloquine, artesunate, and di-hydroartemisinin from 2000 to 2010. Red lines indicate in vitro sensitivity of *P. falciparum* isolates to (A) mefloquine, (B) artesunate, and (C) dihydroartemisinin. Bars indicate 95% CIs. No data were available for 2011.

The in vitro sensitivity (IC_50_) of *P. falciparum* isolates to mefloquine, artesunate, and dihydroartemisinin is shown in Figure 8. There were no specific trends over the study period.

### Climate

**Figure 9:**
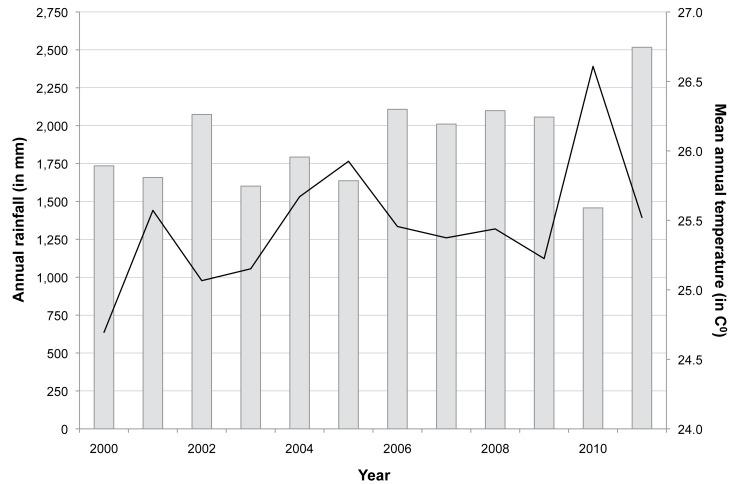
Annual rainfall and mean annual temperature between 2000 and 2011. Black line indicates mean annual temperature in degrees Celsius, and grey bars indicate annual rainfall in millimetres.

The annual rainfall and the mean annual temperature are shown in Figure 9. There was a steady increase in the mean annual temperature of 0.1°C between October 1999 and September 2011, but expected seasonal variations in temperature and rainfall. Over the study period, the mean annual rainfall ranged from 1,600 mm to 2,600 mm. The mean temperature ranged from 22.2°C in December to 28.3°C in April, and annual relative humidity was above 75%. The start of the rains was delayed in 2010, resulting in an overall reduction of annual mean rainfall by half. However, rainfall increased again in 2011.

## Discussion

Since 2000 the transmission of *P. falciparum* has declined substantially in the population in Myanmar living on the border with Thailand in Tak province (the most populated part of the border, with intense migration), despite being a well-known focus of highly drug-resistant *P. falciparum* malaria and an area of documented emergence of *P. falciparum* resistance to artesunate [4]. The decline is evident from the number of cases detected and treated in the SMRU clinics situated on the Thai side of the border. Longitudinal studies in a cohort of pregnant women confirm this decline. The decline has mainly been attributed to improved access to early detection of malaria and treatment with highly effective artemisinin-based antimalarial therapies.

This approach used by SMRU as well as other health providers in the area had proven effectiveness first in the refugee camps on the Thai border in the 1990s, then in Thai villages of Tak province and elsewhere in the region [14], and even in low transmission areas of Africa [15]. This effectiveness is explained by the rapid killing of sexual stages of *P. falciparum* and the activity of artemisinins on gametocytes [16].

Expanding EDT to the mobile and migrant population on the Myanmar side of the border also appears to have been successful in reducing malaria transmission, as evidenced by the decrease in the number of consultations for malaria and the results of the cross-sectional prevalence surveys. The financial investment was relatively modest, consisting mainly of drug and diagnostic test provision.

There are other factors that may have contributed to this reduction in the transmission of *P. falciparum*. There was a sharp decline in rainfall in 2010, and this may have contributed to lower vector abundance. However, the levels of precipitation in 2011 were higher than average, and this finding did not translate into a significant rise in caseload. In general, there was not much seasonal climatic variation from year to year over the period of study, so we do not believe that climate was a major confounding factor for changes in malaria prevalence or incidence observed over time (see detailed climate information in Figure S1). Over 46,000 insecticide-impregnated bed nets were also distributed between 2000 and 2011; however, the vectors in this area (*A. minimus*, *A.dirus*, and *A. maculatus*) are forest mosquitoes feeding early in the evening and outdoors, such that the impact of impregnated nets is limited [17]. These vector characteristics also explain the high proportion of cases in young adult males, because they are exposed while working in the forested areas. Vector control measures would also not explain the differences observed between *P. falciparum* and *P. vivax* case reductions. The latter species has become dominant, and the ratio of *P. falciparum* to *P. vivax* infections has declined in all areas where the patients had access to EDT (Figure 6). The slow decline in the number of *P. vivax* infections may be explained by the decline in *P. falciparum* infections, as many *P. vivax* parasitaemic episodes are relapses from previous infections, and *P. falciparum* infection is associated with *P. vivax* relapse [18]. So the reduction in the number of clinical episodes of *P. falciparum* may have contributed to lower numbers of *P. vivax* relapses [19].

Antimalarial drugs are the cornerstone of EDT, and this situation puts the parasite population under selection pressure for resistance. Indeed, we have shown that the proportion of patients with multiple copies of the gene *Pfmdr1* (known to mediate resistance to mefloquine) is increasing, which may have contributed to the decline in the in vivo efficacy of the MAS_3_ combination recently reported, although the number of participants recruited in 2011 was very small (*n* = 32) [10]. If this sharp drop in efficacy is confirmed, an alternative ACT will have to be registered and deployed. The effectiveness of the current treatment for failures (7 d of quinine and tetracyclines) is poor. More worryingly, resistance to artemisinin is also emerging. This situation translates into a reduction of the parasite clearance rate following treatment with artesunate and may be explained by parasite genetics [20]. Unfortunately, resistance to artemisinin is not detected by conventional in vitro susceptibility testing methods, as shown in this study. The observation of increasing gametocyte carriage in patients presenting with *P. falciparum* infection is of great concern. The mechanisms that confer resistance to artemisinin are unknown, but it has been suggested that the use of substandard drugs and monotherapies may be contributing factors [3]. However, in the border population described here, self-treatment and monotherapies are uncommon. Alternatively, the use of quality drugs, free of charge, even in combination, may have selected parasite clones resistant to artesunate. This situation does not question the rationale behind using ACTs [16] but may be related to the phenomenon of dormancy [21]. When ring stages of *P. falciparum* are exposed to artesunate (or its metabolite, dihydroartemisinin), a small proportion becomes metabolically inactive and insensitive to drugs, and these are not removed by the spleen, unlike those affected by the drug [22] and unlike what is normally seen in patients [23],[24]. Whether the two observations (dormancy and the slow clearance phenotype) are related is unknown but cannot be excluded [25],[26]. To complicate matters further, *P. vivax* is becoming more resistant to chloroquine in this area [27], and this finding may explain the increasing proportion of patients presenting with vivax gametocytes (Figure 2). It may be necessary to adopt a unified ACT for both species, as in Papua province, Indonesia [28].

The observational nature of some of these data is an obvious limitation, and there are potential biases. The unstable nature of the population and the difficulties of working across an international border mean that the cases detected may not be representative of the overall population. Pregnant women, the group in whom we defined malaria incidence, may have a different risk of exposure and may not reflect the disease burden in the general population. However, it should be acknowledged that in most malarious regions (in particular in border areas), data are either nonexistent or much less detailed than in this study. We cannot eliminate all sources of bias, but the decline in *P. falciparum* cases in all age groups and both genders remains robust.

The benefit of increased access to EDT for the population is obvious, as illustrated by the decrease in maternal malaria-related anaemia. Efforts are underway to try to contain the spread of artemisinin resistance from western Cambodia. Our results and results from elsewhere indicate that an aggressive strategy based on EDT of cases, combined with vector control and information to the population, is the way forward to eliminate malaria. However, it is uncertain whether this strategy alone could achieve complete elimination over time. Adjunctive approaches should be evaluated, such as the use of low-dose primaquine to reduce transmission further, mass drug administrations, more sensitive detection of sub-microscopic infections using molecular methods, or targeted use of the new malaria vaccine, if it is approved. Addition of all these interventions may not be cost-effective, and so this approach should also be assessed.

Considerable support and strong leadership are urgently needed to step up the malaria control program in Myanmar and elsewhere. In practice, this means increasing the population coverage and training of village health workers in the use of RDTs and ACTs plus primaquine. Eliminating *P. falciparum* or dramatically reducing the number of cases is feasible, but the main obstacle is the difficulty of accessing populations living in remote, sometimes dangerous areas and across international borders.

## Supporting Information

Figure S1 **Monthly mean temperature in districts of Tak province bordering Myanmar.**
(PDF)

## Abbreviations

ACT: artemisinin-based combination treatment
EDT: early diagnosis and treatment
MAS_3_: mefloquine-artesunate
QC: quality control
RDT: rapid diagnostic test
SMRU: Shoklo Malaria Research Unit
