# Supplementary figures and images for: Malaria Burden and Artemisinin Resistance in the Mobile and Migrant Population on the Thai–Myanmar Border, 1999–2011: An Observational Study
Source: PLoS Med. 2013 Mar 5;10(3):e1001398. doi: 10.1371/journal.pmed.1001398 (PMC3589269; doi:10.1371/journal.pmed.1001398)

## Monthly mean temperature (in C°) in districts of Tak province bordering Myanmar

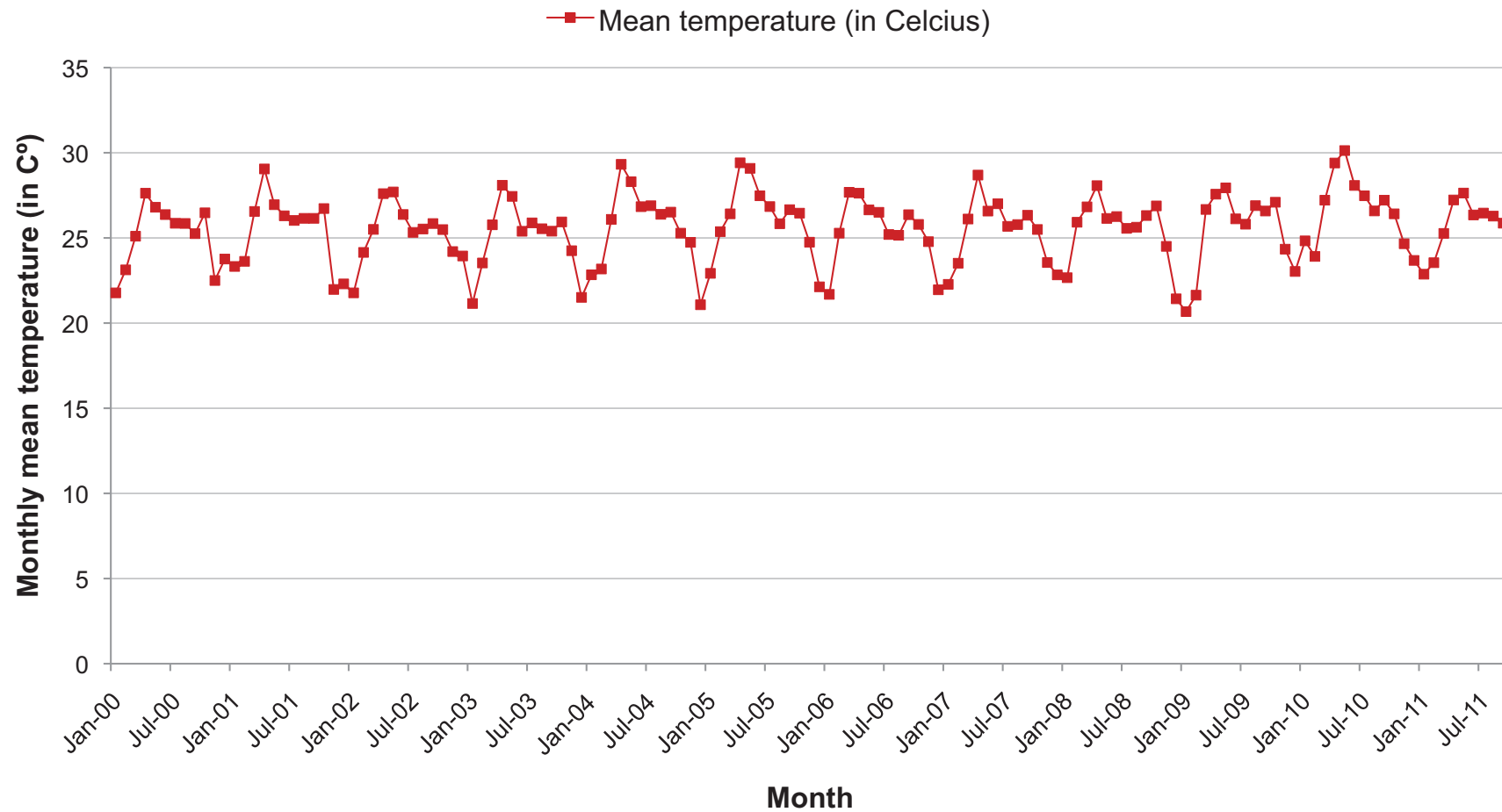

Supplement: Figure S1 — Monthly mean temperature in districts of Tak province bordering Myanmar. (PDF) [file pmed.1001398.s001.pdf]
